# Cascade and no‐repetition rules are comparable controls for the auditory frequency mismatch negativity in oddball tasks

**DOI:** 10.1111/psyp.13280

**Published:** 2018-09-24

**Authors:** Stefan Wiens, Malina Szychowska, Rasmus Eklund, Erik van Berlekom

**Affiliations:** ^1^ Gösta Ekman Laboratory, Department of Psychology Stockholm University Stockholm Sweden

**Keywords:** cascade, mismatch negativity, N1, neural adaptation, no‐repetition, oddball

## Abstract

The mismatch negativity (MMN) has been widely studied with oddball tasks to index processing of unexpected auditory change. The MMN is computed as the difference of deviant minus standard and is used to capture the pattern violation by the deviant. However, this oddball MMN is confounded because the deviant differs physically from the standard and is presented less often. To improve measurement, the same tone as the deviant is presented in a separate condition. This control tone is equiprobable with other tones and is used to compute a corrected MMN (deviant minus control). Typically, the tones are in random order except that consecutive tones are not identical (no‐repetition rule). In contrast, a recent study on frequency MMN presented tones in a regular up‐and‐down sequence (cascade rule). If the cascade rule is detected more easily than the no‐repetition rule, there should be a lower risk of a confounding MMN within the cascade condition. However, in previous research, the cascade and no‐repetition conditions differed not only in the regularity of the tone sequence but also in number of tones, frequency range, and proportion of tones. We controlled for these differences to isolate effects of regularity in the tone sequence. Results of our preregistered analyses provided moderate evidence (BF_01_>6) that the corrected MMN did not differ between cascade and no‐repetition conditions. These findings imply that no‐repetition and cascade rules are processed similarly and that the no‐repetition condition provides an adequate control in frequency MMN.

## 1 INTRODUCTION

In everyday life, our immediate surroundings change constantly. Because these changes may be goal relevant, they need to be detected, especially if they are unexpected. In the auditory domain, the mismatch negativity (MMN) has been widely used to index processing of unexpected auditory change (Fishman, 2014; Kujala, Tervaniemi, & Schröger, 2007; Näätänen & Kreegipuu, 2011; Näätänen, Gaillard, & Mantysalo, 1978; Winkler, 2007). Traditionally, the MMN has been derived from EEG in an oddball task (Duncan et al., 2009). Subjects are presented with a sequence of identical tones (standards) interspersed with rarely occurring different tones (deviants or oddballs). The regular presentation of the standards creates a pattern that is violated by the occasional deviants. The MMN is a difference wave and is obtained by subtracting the average EEG response to the deviants by the average EEG response to the standards (i.e., deviant minus standard). With the nose as the reference for the EEG, the MMN is apparent on the scalp as a frontocentral negativity about 150 to 200 ms after the onset of the deviant. It is typically accompanied by a polarity reversal (i.e., positivity) over the mastoid electrodes.

Unfortunately, the oddball task is confounded because the deviant differs physically from the standard and is also presented less often than the standard. Physical differences have strong effects on the auditory N1, a frontocentral negativity about 100 ms after tone onset (May & Tiitinen, 2010; Näätänen & Picton, 1987). For example, the N1 is larger in response to a loud than a soft tone. Therefore, an apparent frontocentral negativity may actually indicate only effects of these physical differences rather than of an unexpected auditory change.

**Figure 1:**
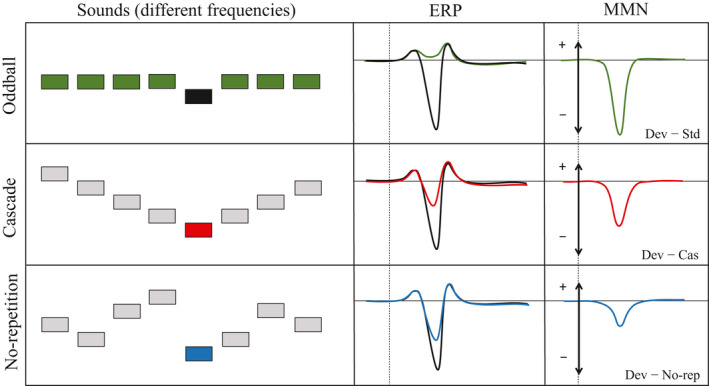
Left: Illustration of sound order in the oddball (top), cascade (middle), and no‐repetition (bottom) conditions. Middle: Schematic ERPs to the deviant (black), standard (green), cascade control (red), and no‐repetition control (blue). Right: Resulting difference waveforms of the traditional oddball MMN (top), cascade‐corrected MMN (middle), and no‐repetition‐corrected MMN (bottom)

To control for these confounding effects, Schröger and Wolff (1996) suggested a separate control condition in which the same tone as the deviant is presented as many times as the deviant is presented in the oddball condition, and this control tone is equiprobable with other tones. For example, if the deviant is presented on 10% of the trials in the oddball condition, the control tone is presented on 10% of the trials and is equiprobable with nine other tones in the control condition. A corrected MMN difference wave is obtained by taking the response to the deviant in the oddball condition minus the response to the control tone in the control condition (i.e., deviant minus control). Because the deviant in the oddball task is physically identical to the control tone and both tones are presented equally often, any obvious confounding effects from N1 differences are avoided. This condition has been introduced to study pattern violations in frequency (Jacobsen & Schröger, 2001; Jacobsen, Schröger, Horenkamp, & Winkler, 2003), location (Schröger & Wolff, 1996), duration (Jacobsen & Schröger, 2003), and intensity (Jacobsen, Horenkamp, & Schröger, 2003). In these studies, trial order was pseudorandomized but complied with a no‐repetition rule so that tone repetitions were avoided. Below, we refer to this control condition as the no‐repetition condition. Figure 1 illustrates how the oddball MMN and the no‐repetition‐corrected MMN are calculated.

Unfortunately, the no‐repetition condition may not be an ideal control condition. One concern is that it may yield a smaller MMN on the scalp and thus underestimate the response to unexpected auditory change (Jacobsen & Schröger, 2001; Ruhnau, Herrmann, & Schröger, 2012; Schröger, 2007; Schröger & Wolff, 1996). When a tone is repeated or is followed by other tones that are tonotopically close, the N1 decreases because of neural adaptation (May & Tiitinen, 2010). In the oddball task, the deviant and standard are tonotopically close, whereas in the no‐repetition condition, the control tone and the other tones are tonotopically farther away. Therefore, the response to the control tone shows less neural adaptation than that to the deviant. Consequently, the N1 is larger to the control tone than the deviant, and this enlarged N1 to the control tone would lead to a smaller corrected MMN difference of deviant minus control. However, if an MMN is observed despite this confound, it may be hard to account for it in terms of neural adaptation (but see May & Tiitinen, 2010).

Another concern is that an optimal control condition should have a clear pattern that is not violated (Ruhnau et al., 2012). Notably, previous research suggests that the auditory system detects even complex tone patterns (Paavilainen, 2013). For example, when subjects watched silent movies, an MMN was found with magnetoencephalography (MEG) to violations of three‐tone patterns (Kuchenbuch, Paraskevopoulos, Herholz, & Pantev, 2013), of four‐tone to eight‐tone patterns (Boh, Herholz, Lappe, & Pantev, 2011), and of two simultaneous five‐tone patterns (Fujioka, Trainor, Ross, Kakigi, & Pantev, 2005). In regard to the no‐repetition rule, research suggests that it is detected because violations of the rule elicit an MMN (for a review, see Horváth & Winkler, 2004). However, given the pseudorandom order of the tones, it is unclear how easily the no‐repetition rule is detected. For example, if the no‐repetition rule is difficult to detect, the pseudorandom order might result in local patterns that, if violated, might yield a confounding MMN (Winkler et al., 1990). In response, Ruhnau et al. (2012) proposed the cascade rule: Tones are presented in a regular sequence of ascending and descending frequencies over consecutive tones. Because the tones in the cascade condition represent a clear pattern, there should be no risk of a confounding MMN within the cascade condition because the pattern is never violated. Thus, the difference between deviant and control tone from the cascade condition should be a cleaner measure of the corrected MMN than the difference between deviant and control tone from the no‐repetition condition. Figure 1 illustrates this hypothesis: The cascade‐corrected MMN (i.e., deviant minus cascade) should be larger than the no‐repetition‐corrected MMN (i.e., deviant minus no‐repetition).

In the study that introduced the cascade rule, Ruhnau et al. (2012) recorded N1 and MMN during three conditions: oddball, cascade, and no‐repetition (which the authors refer to as random). If the cascade rule is detected more easily than the no‐repetition rule, the risk of a confounding MMN within the conditions should be smaller for the cascade than for the no‐repetition condition. Accordingly, the mean amplitudes in the MMN‐relevant interval should be less negative in the cascade than no‐repetition condition, yielding a larger (i.e., more negative) corrected MMN (i.e., deviant minus control) in the cascade than no‐repetition condition (Figure 1). Results for frontal‐central electrodes showed that the mean amplitudes for the corrected MMN did not differ significantly between the conditions. In contrast, the mean N1 amplitudes were significantly smaller (i.e., amplitudes were less negative) in the cascade than no‐repetition condition. Because Ruhnau et al. provided us with their preprocessed data, we were able to conduct secondary analyses to perform Bayesian hypothesis testing (Wiens, Szychowska, Eklund, & van Berlekom, 2018). These analyses provided only anecdotal evidence with regard to condition differences in the corrected MMN.

Critically, the cascade and no‐repetition conditions in the Ruhnau et al. (2012) study differed in several aspects over and above the differences in regularity of the tone sequence. Relative to the no‐repetition condition, the cascade condition included fewer frequencies with a smaller frequency range, and the neighboring tones to the control tones were presented twice as often. These differences in combination have been shown to decrease both MMN and N1 (Jacobsen, Schröger, et al., 2003). In their study, Jacobsen et al. compared the deviant tone in an oddball condition to matched control tones in different contexts. These contexts combined manipulations of three parameters: First, the number of other tones aside from the control tone decreased from nine to two in the different contexts. Second, the frequency range decreased from 629 to 55 Hz. Third, the proportion of these other tones increased from 10% to 45%. Results showed that when there were few other tones with a small frequency range in a large proportion (similar to the cascade condition), the corrected MMN (i.e., deviant minus control) decreased. Apparently, when the control tone was relatively rare among other tones, it elicited an MMN by itself because it was a deviant relative to the other tones (Winkler et al., 1990). As a result, the corrected MMN was reduced.

Results also showed that the same combined manipulation decreased the N1 to the control tone (Jacobsen, Schröger, et al., 2003). In further support, another study on the N1 presented tones at eight different frequencies in no‐repetition conditions, and the eight tone frequencies had either a small overall frequency range with small spacing between frequencies or a large overall frequency range with large spacing between frequencies (Herrmann, Henry, & Obleser, 2013). Results showed that the N1 to the edge stimuli (which correspond to control tones) were smaller when the overall frequency range (and the spacing between frequencies) was small rather than large. Accordingly, the N1 in the Ruhnau et al. (2012) study may have been smaller in the cascade than no‐repetition condition because of neural adaptation, as the other tones were tonotopically closer to the control tone and were presented more often. Therefore, one cannot resolve whether the results obtained by Ruhnau et al. are caused by differences in regularities or by these potential confounds.

In the present study, the cascade and no‐repetition conditions were identical in terms of number of tones, their frequency range, and their relative proportions. They differed only in terms of the regularity in the tone sequence. Therefore, the present study isolates the effects of regularity from potential confounds.

## 2 METHOD

Method and analyses were preregistered in detail before any data were collected (osf.io/ctx69). Deviations from the preregistration are noted below. All data and scripts are available via figshare (Wiens et al., 2018).

### 2.1 Participants

The initial sample consisted of 40 healthy subjects (*M* = 25.5 years, *SD* = 5.6). Seventeen were male, and 36 were right‐handed. Subjects had normal or corrected‐to‐normal vision and were recruited from local universities and through online billboards. They were compensated with either movie vouchers or course credits after their participation. Subjects provided written consent in accordance with the Declaration of Helsinki. The research was conducted in accordance with the principles of the regional ethics board. Subjects were excluded according to our preregistered criteria (for each subject, we note only the main reason of exclusion). First, subjects were screened with pure tone audiometry (at 500, 750, and 1,000 Hz) to ensure normal hearing (≤20 dB HL). Second, subjects (*n* = 2) were excluded because of poor visual detection performance. For each block and subject, we computed the absolute difference between actual and counted plus signs. If the maximum difference for a subject was 2 *SD*s above the mean across subjects, the subject was excluded. Third, subjects (*n* = 10) with no clear auditory N1 were excluded. This decision was a consensus vote of three authors when viewing the individual ERPs across the standard trials from the oddball condition (that were independent from the ERPs in the primary analyses). The N1 per se was defined by the grand mean wave across all subjects. Fourth, subjects (*n* = 2) were excluded because, after artifact rejection, fewer than 70% trials remained for an ERP in the primary analyses. The final sample comprised 26 subjects. For completeness, we also report the main results for all 40 subjects. Because the peak latencies of N1 and MMN were almost identical for the final and complete samples (see below), we used the peak latencies defined for the final sample in all analyses. Because the main results were similar for the final and complete samples, these results suggest that our preregistered exclusion criteria were unnecessarily strict.

### 2.2 Apparatus

Auditory stimuli were tones at 500, 550, 605, 666, and 732 Hz (100‐ms duration at 70 dB SPL). Visual stimuli were a small circle and a plus sign (0.1°), and the letters were *X, H, K, M, N, V, W*, and *Z*. Letter rings consisted of either six letters or one letter and five small circles. Letter height was 0.4°, and the letters were presented at an eccentricity of 1.7°. A Cedrus StimTracker (Cedrus Corporation, San Pedro, CA) was used to detect and mark the onset of the tones with TTL triggers from the audio output. The onset of the letter rings was also marked with a photodiode in the corner of the screen (where a white square was shown at the same time as the letter ring).

### 2.3 Procedure

Subjects performed a visual detection task on a small circle in the middle of the screen. On each 500‐ms trial, the circle was shown in the middle of the screen and was surrounded by a ring of six letters (the circle and ring were shown for 100 ms). A 100‐ms tone was presented at the same time. Subjects were instructed to attend to the circle and ignore the letters and the tone. They were to count the number of trials in which a plus sign rather than a circle was shown. Each block consisted of 360 trials (360 × 0.5 s = 3 min), and there was a plus (instead of the circle) on 15–20 trials, randomly determined for each block (after a plus, there were at least 6 trials with a circle). After each block, subjects wrote down the number of plus signs that they counted.

The design was repeated measures with two independent variables: tone (oddball, cascade, and no‐repetition) and ring (one or six letters). Each block tested one of the six combinations of tone and ring. For each subject, these six conditions were presented twice, and within each set of six, condition order was randomized. In the present study, only the tone manipulation is of interest whereas the ring manipulation is irrelevant. It was merely included to ensure that the number of letters (one or six) in a task‐irrelevant letter ring does not affect N1 and MMN. This question is relevant for studies in which the letter ring was task relevant and was used to manipulate perceptual load (Szychowska, Eklund, Nilsson, & Wiens, 2017; Wiens, Szychowska, & Nilsson, 2016). We note that, whereas the preregistration uses the term load, we refer to ring instead because load is misleading in the present context.

The three tone conditions were as follows: In the oddball condition, a 500‐Hz tone (deviant) was presented on 1/8th (12.5%) of the trials and a 550‐Hz tone (standard) on the remaining trials. For each block, tone order was random with the restriction that there were at least three standards before the next deviant. Specifically, the distribution of the interdeviant intervals was symmetric and normal (*M* = 7, *SD* = 2, minimum = 3, maximum = 11). At the beginning of each block, seven additional standard tones were presented (but excluded in the data analysis). In the cascade condition, the frequency of the tones over trials increased and decreased in a fixed order (500, 550, 605, 666, 732, 666, 605, 550 Hz), and this set was repeated throughout the block. Before the occurrence of the first 500‐Hz tone, one set of tones without the 500‐Hz tone was presented (but excluded in the data analysis). In the no‐repetition condition, the tones were the same as in the cascade condition (500, 550, 605, 666, 732, 666, 605, 550 Hz), but the order of the tones was pseudorandomized within each set so that the no‐repetition rule would apply both within and between consecutive sets. At the beginning of each block, an additional set of tones without the 500‐Hz tone was presented in a similar pseudorandom order (but excluded in the data analysis).

The two ring conditions were as follows: In the one‐letter condition, the letter ring consisted of one letter and five small circles. On half of the trials, the letter was *X*, and on the other half, the letter was chosen randomly from the set of *H, K, M, N, V, W,* and *Z*. The clock position of the letter was determined randomly (at positions 2, 4, 6, 8, 10, and 12 o'clock). The order of trials with an *X* or without an *X* was random with the restriction that there could be no more than three trials of either type in a row. In the six‐letter condition, the letter ring consisted of six letters chosen from *X, H, K, M, N, V, W, *and *Z*. On each trial, the six letters were drawn randomly without replacement with the restriction that the letter *X* was present on half of the trials (at a random clock position). The order of trials with an *X* or without an *X* was random with the restriction that there could be no more than three trials of either type in a row.

### 2.4 EEG recording and analysis

EEG data were recorded from six electrodes at standard 10/20 positions (Fpz, Fz, FCz, Cz, P9, P10) and two additional electrodes (tip of nose, one on the cheek) with an ActiveTwo BioSemi system (BioSemi, Amsterdam, Netherlands). Fpz, Fz, FCz, Cz, P9, and P10 were recorded with pin electrodes in a 64‐electrode EEG cap, and the tip of the nose and the cheek were recorded with flat electrodes attached with adhesive disks. Because the left and right mastoids (M1 and M2) were not available in the EEG cap, we used the nearby positions P9 and P10 for convenience. Two additional system‐specific electrodes were recorded with pin electrodes in the EEG cap. The common mode sense (CMS; between PO3 and POz) served as the internal reference electrode, and driven right leg (DRL; between POz and PO4) as the ground electrode. Data were sampled at 1,024 Hz and filtered with a hardware low‐pass filter at 104 Hz and a software high‐pass filter at 0.1 Hz. The EEG data were processed further with MATLAB scripts and the toolbox FieldTrip (Oostenveld, Fries, Maris, & Schoffelen, 2011).

Epochs were extracted from 100 ms before tone onset to 400 ms after. Fpz, Fz, FCz, Cz, and the mastoids were referenced to the tip of the nose, and Fpz was also referenced to the cheek electrode (for vertical and horizontal electro‐oculogram). Each epoch was baseline corrected with the mean of the 100‐ms interval before tone onset. For each participant, amplitude ranges (i.e., max minus min) within individual epochs were extracted, and the distribution of these was visually inspected to exclude apparent outliers. Cutoffs were adjusted individually to retain as many trials as possible while reducing the potential effects of outliers. Inspection was blind to the condition (tone and ring) of individual trials and, thus, this inspection avoided bias (Keil et al., 2014). To assess effects of this visual inspection on actual amplitude ranges, we computed the amplitude ranges on individual trials for the main conditions (i.e., deviant and standard tones in the oddball condition and control tones in the cascade and no‐repetition conditions) and the main electrodes (i.e., Fz, FCz, Cz). For each subject, we computed the maximum amplitude range across these trials. For the final sample (*N* = 26), the mean of the maximum amplitude range was 139.6 µV, and no subject had a maximum amplitude range that exceeded 194.9 µV. For the complete sample (*N* = 40), the mean of the maximum range was 138.3 µV, and no subject had a maximum amplitude range that exceeded 225.8 µV.

Mean ERP amplitudes were extracted for N1‐relevant and MMN‐relevant intervals. The intervals were not predefined but were derived from the trials in the oddball condition, as described below. Critically, this data‐driven procedure did not bias any results for N1 and MMN between the cascade and no‐repetition conditions, as the definitions of N1 and MMN were independent from these data. To visually identify the auditory N1, a mean wave was computed across standard trials from the oddball condition (across both ring conditions and subjects). This mean wave was low‐pass filtered at 30 Hz. The N1 was defined as the peak negativity across Fz, FCz, and Cz at approximately 100 ms. After identifying the N1 across all subjects (apparent at 89 ms), we visually inspected individual N1 waves. As defined in the preregistration, if a subject did not show an N1, the subject was excluded from the sample (*n* = 10). For the final sample (*n* = 26), the N1 peak was redefined (apparent at 93 ms), and for each subject, mean N1 amplitudes (across Fz, FCz, Cz) were computed for a 30‐ms interval (center of peak ±15 ms), separately for the six conditions.

To visually identify the auditory MMN, a difference wave was computed for each subject by subtracting the mean ERP to standards from that to deviants in the oddball condition (across both ring conditions). This difference wave was averaged across Fz, FCz, and Cz and across subjects, and was low‐pass filtered at 30 Hz. This difference wave showed an apparent negativity between 100 and 250 ms after tone onset with a concurrent polarity reversal (i.e., positivity) over the mastoids. For the interval defined by the peak of this negativity (140 ± 25 ms), mean amplitudes were extracted separately for each of the six conditions and also for the standard tones in the oddball condition. For the complete sample, the peak of the negativity was at 141 ms.

In hindsight, it may have been preferable to define the auditory N1 from the mean wave across the other tones (i.e., 605, 666, 732 Hz) in the cascade and no‐repetition conditions rather than from the mean wave across the standard trials. Whereas the N1 to the standards may be difficult to identify because of neural adaptation, the N1 to the other tones would be large and would remain independent from the main analyses of the 500‐Hz tone. However, as apparent in the figures of the grand means (see below), the standards adequately captured the timing of the N1.

Furthermore, it is possible that our preregistered criterion to exclude subjects without an N1 to standards may have biased the results. Critically, as described below, results were unaffected by whether or not we excluded subjects. Therefore, we report the analyses according to our preregistered N1 criterion.

### 2.5 Statistical analyses

We conducted Bayesian hypothesis testing to determine the degree of evidence for or against the alternative hypothesis (Dienes, 2014; Wagenmakers, Marsman, et al., 2017; Wiens & Nilsson, 2017). The Bayes factor (BF) expresses the likelihood of the data given the alternative hypothesis (i.e., theoretical predictions) relative to the likelihood of the data given the null hypothesis. Simply put, it captures how much better the data are explained by the alternative hypothesis versus the null hypothesis. The better the data are explained by one rather than the other hypothesis, the more evidence there is in support of this hypothesis. For example, a BF_10_ = 3 means that there is three times more evidence for the alternative than the null hypothesis, whereas a BF_01_ = 3 means that there is three times more evidence for the null than the alternative hypothesis.

As preregistered, the alternative hypothesis was that the N1 amplitude in the cascade condition would be smaller (i.e., less negative) than the N1 amplitude in the no‐repetition condition. For cascade, no‐repetition, and oddball conditions, the mean N1 amplitudes were computed for the 500‐Hz tones across the ring conditions. Because we computed the amplitude difference of cascade minus no‐repetition, a positive difference score would support the hypothesis that the N1 was smaller (i.e., less negative) in the cascade than no‐repetition condition. Furthermore, the N1 to the deviant in the oddball condition should be smaller than the N1 to the control tones. Because in the oddball condition deviant and standard tones are tonotopically close to each other, the N1 to the deviant should be decreased because of neural adaptation. In contrast, because in the cascade and no‐repetition conditions the tones within each condition are tonotopically further away from each other, the N1 to the control tones should be less decreased because of neural adaptation. As a consequence, the N1 difference between cascade and no‐repetition control tones should not be larger than the N1 difference between deviant and no‐repetition control tone. Because a small N1 to the deviant minus a large N1 to the no‐repetition control should give a large positive value, the alternative hypothesis was modeled as a uniform distribution with the lower limit defined as zero and the upper limit defined as the N1 difference of deviant minus no‐repetition control. The null hypothesis was that the N1 difference of cascade minus no‐repetition would be zero. Because, in principle, the BF compares two models with each other, we conducted exploratory analyses with other alternative hypotheses to assess if the findings are robust or vary depending on the alternative hypothesis (Dienes, 2014).

In regard to the MMN, the alternative hypothesis was that the cascade‐corrected MMN (deviant minus cascade) would be larger (i.e., more negative) than the no‐repetition‐corrected MMN (deviant minus no‐repetition). Because we computed (deviant minus no‐repetition) minus (deviant minus cascade), a positive difference score implies that the cascade‐corrected MMN was larger (i.e., more negative) than the no‐repetition‐corrected MMN. The mean amplitudes that were relevant for the MMN were computed for the 500‐Hz tones across the ring conditions. For the BF, the alternative hypothesis was modeled as a uniform distribution with the lower limit defined as zero and the upper limit defined as the absolute size of the oddball MMN (deviant minus standard) to obtain a positive upper limit. The null hypothesis was that the difference of no‐repetition‐corrected MMN minus cascade‐corrected MMN would be zero. We also conducted exploratory analyses with other alternative hypotheses.

In addition, we tested if the effects above varied with ring condition (one or six letters). We predicted no effects. Because results were inconclusive, they are not reported below but are available via figshare (Wiens et al., 2018).

The BF was calculated with Aladins Bayes factor in R (Wiens, 2017). These scripts compute and plot the BF for mean differences in raw units if the alternative hypothesis is modeled as a normal, *t*, or uniform distribution, and the likelihood is modeled as a normal or *t* distribution (Dienes & McLatchie, 2018). In the present analyses, the alternative hypotheses were modeled as uniform distributions and the likelihoods as *t* distributions. Although the BF is a continuous measure of evidence, we adopted a common interpretation scheme (Wagenmakers, Love, et al., 2017). For example, 1 < BF < 3 is anecdotal evidence, and 3 < BF < 10 is moderate evidence.

## 3 RESULTS

On average, subjects in the final sample did not miscount the number of plus signs (between 15 and 20 per block) by more than 5.05 (*SD* = 1.04).

**Figure 2:**
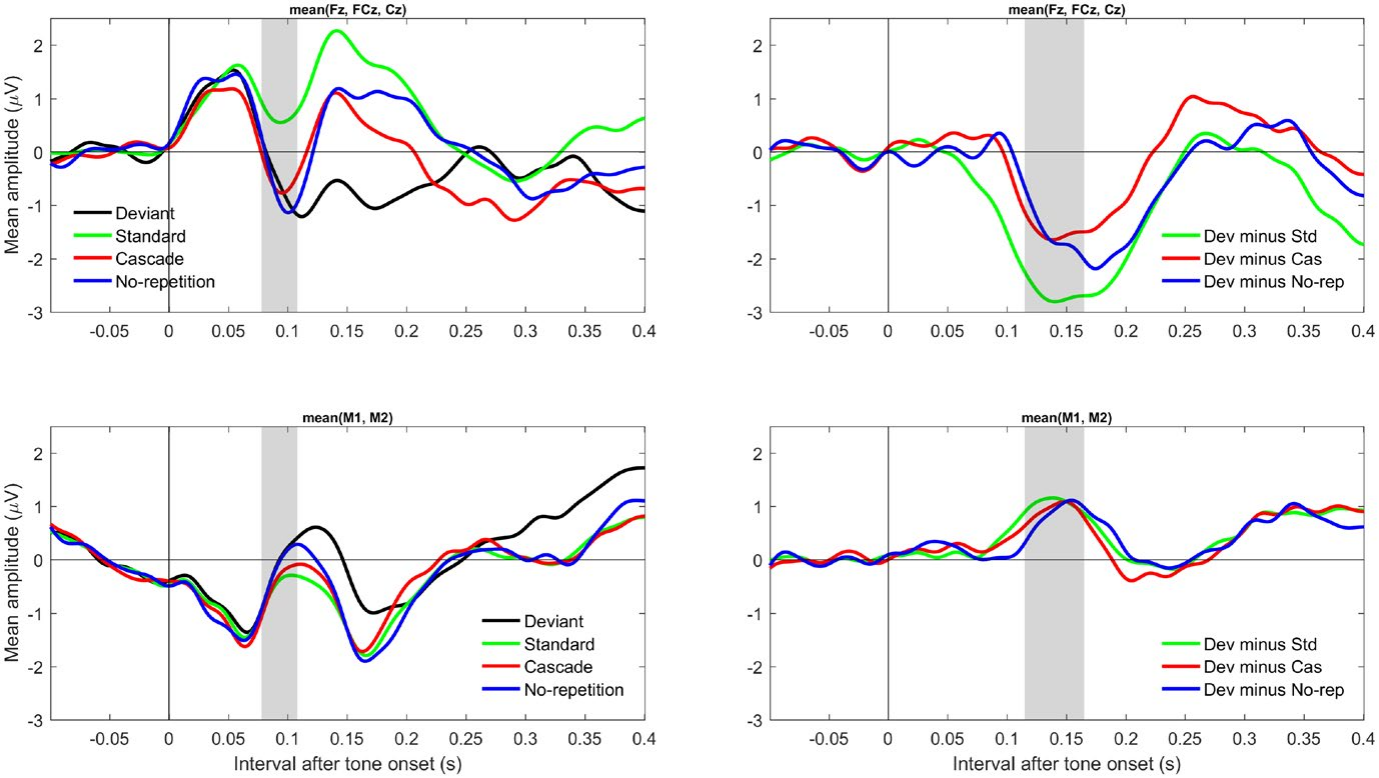
Grand‐averaged (*N* = 26) ERPs (left column) and difference waves (right column) to the tone onset across frontocentral electrodes (upper row) and mastoids (lower row). The gray bars mark the N1‐relevant interval (left column) and the MMN‐relevant interval (right column). The data were low‐pass filtered at 30 Hz

Figure 2 shows the grand‐averaged ERPs and difference waves to the tone onset across frontocentral electrodes (upper row) and mastoids (lower row). Note that a similar figure across all 40 subjects (available via figshare) looks almost identical to Figure 2, showing that excluding subjects did not bias the results. The left column suggests that the N1 (gray bar) was comparable among deviant, cascade, and no‐repetition tones but was smaller (i.e., less negative) to standard tones. The right column suggests that the oddball MMN (deviant minus standard) was larger (i.e., more negative) than the corrected MMN (deviant minus control). Notably, the corrected MMN appeared similar for the cascade and no‐repetition conditions.

For the N1‐relevant interval (78–108 ms), mean amplitudes for frontocentral electrodes were 0.66 for standard, −0.55 for deviant, −0.58 for cascade, and −0.72 µV for no‐repetition tones. The mean amplitude difference of cascade minus no‐repetition tones was 0.14, 95% CI [−0.36, 0.64], BF_01_ = 0.88. Note that for the complete sample (*n* = 40), *M* = 0.12, 95% CI [−0.34, 0.57], BF_01_ = 0.96. For these BF analyses, the alternative hypothesis was defined to range from zero to the difference score of deviant minus no‐repetition (e.g., for the final sample: −0.55 minus −0.72 = 0.17 µV). These results provide inconclusive evidence for or against differences in N1 between cascade and no‐repetition conditions.

However, other alternative hypotheses are conceivable: First, although the deviant should elicit a small N1 due to neural adaptation, it may be biased toward a larger N1 by an early MMN effect. If so, the N1 to the deviant would be larger (i.e., more negative) than it should be. Unfortunately, it is difficult to estimate the size of an early MMN effect. Although the standard does not reflect an MMN effect, its N1 should be particularly low due to neural adaptation and, thus, it is biased toward a small N1. Nonetheless, we explored effects of redefining the upper limit of the alternative hypothesis as the N1 difference of standard minus no‐repetition control (0.66 minus −0.72 = 1.38 µV). With this wide alternative hypothesis, the BF_01_ = 2.63 in the final sample (and BF_01_ = 2.70 in the complete sample) starts to provide anecdotal evidence for no difference in N1. Indeed, the vaguer the alternative hypothesis, the more the BF favors the null hypothesis; thus, the BF punishes vagueness (Dienes & McLatchie, 2018). Second, the frequency steps between consecutive tones were more variable in the no‐repetition than cascade condition. Because effects of this frequency variability on the N1 are unclear, we performed a two‐tailed analysis in which the upper limit of the alternative hypothesis was defined as standard minus no‐repetition control and the lower limit as its inverse (e.g., for the final sample, the alternative hypothesis was defined to range between −1.38 µV and 1.38 µV). With this even vaguer alternative hypothesis, the BF_01_ = 3.70 in both the final and complete samples provides moderate evidence for no N1 differences between cascade and no‐repetition conditions. These results suggest that, at best, the present data provide moderate evidence for no condition differences in N1.

**Figure 3:**
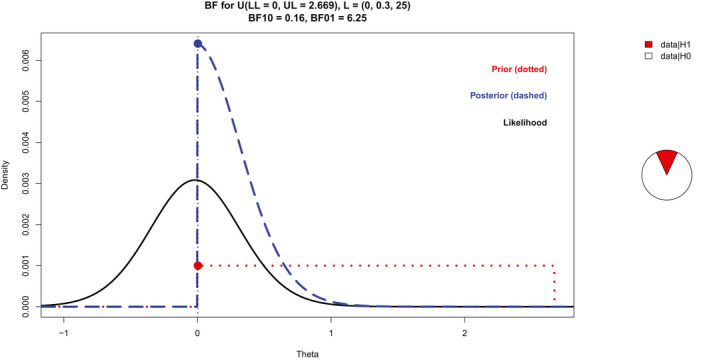
Plot of the Bayes factor (BF) for the mean amplitude difference between no‐repetition‐corrected MMN (deviant minus no‐repetition) and cascade‐corrected MMN (deviant minus cascade). It illustrates the prior (i.e., the alternative hypothesis), likelihood, and posterior. For the prior, the difference of no‐repetition‐corrected MMN minus cascade‐corrected MMN was assumed to be uniform between zero and no larger than the absolute size of the oddball MMN (deviant minus standard). Thus, a positive theta (in µV) reflects a larger corrected MMN (deviant minus control) in the cascade than no‐repetition condition. The white area dominates the pie chart, showing that the data support the null hypothesis six times more than the alternative hypothesis

For the MMN‐relevant interval (115–165 ms), mean amplitudes for frontocentral electrodes were 1.91 for standard, −0.76 for deviant, 0.74 for cascade, and 0.76 µV for no‐repetition tones. Relative to the standard in the oddball condition, the other tones were negative. Specifically, there was an oddball MMN (deviant minus standard), *M* = −2.67, 95% CI [−3.30, −2.03], a cascade‐corrected MMN (deviant minus cascade), *M* = −1.50, 95% CI [−2.24, −0.76], and a no‐repetition‐corrected MMN (deviant minus no‐repetition), *M* = −1.52, 95% CI [−2.31, −0.72]. The oddball MMN was larger than the no‐repetition‐corrected MMN, *M* = −1.15, 95% CI [−1.88, −0.42]. Critically, there was no difference between the no‐repetition‐corrected MMN and the cascade‐corrected MMN, as the mean difference was almost zero; *M* = −0.02, 95% CI [−0.72, 0.69]. Note that a positive difference score would have been expected if the cascade‐corrected MMN was larger (more negative) than the no‐repetition‐corrected MMN. Figure 3 shows a plot of the BF for this difference. The BF_01_ = 6.25 implies that the data were six times more likely given the null hypothesis than given the alternative hypothesis. Note that for the complete sample, *M* = 0.15, 95% CI [−0.40, 0.70], BF_01_ = 4.35.

For these preregistered BF analyses of the MMN, the alternative hypothesis was defined as ranging from zero to the absolute size of the oddball MMN (deviant minus standard), for example, from 0 to 2.67 for the final sample. However, other alternative hypotheses may be explored: First, one could argue that the oddball MMN overestimates the size of the true MMN, as the standard is biased toward positivity due to neural adaptation. Unfortunately, the true MMN is unknown, and the cascade‐corrected MMN probably underestimates the true MMN. Nonetheless, if the cascade‐corrected MMN is used to define the upper limit, this narrower alternative hypothesis decreases the BF_01 _= 3.57 in the final sample (BF_01 _= 2.44 in the complete sample). The BF_01 _decreases because as the alternative hypothesis becomes narrower, it is harder to distinguish between the hypotheses (Dienes & McLatchie, 2018). Second, one may argue that there is no strong theoretical reason to think that the corrected MMN should be stronger for the cascade than the no‐repetition rule, and effects may actually be reversed. To conduct a two‐tailed analysis, the alternative hypothesis was modeled to range in either direction to the size of the oddball MMN (e.g., −2.67 to 2.67 for the final sample). The BF_01 _= 6.25 in both the final and complete samples provides moderate evidence for similar effects of the cascade and no‐repetition rule on the corrected MMN.

## 4 DISCUSSION

Two control conditions for the oddball condition were designed to be the same except for adhering to a cascade rule (i.e., up‐and‐down sequence) or a no‐repetition rule. The preregistered analyses suggested that there was moderate evidence (*BF*_01 _> 6) that the corrected MMN (deviant minus control) was similar in both control conditions. With regard to N1, there was no evidence for or against similar effects of the cascade and no‐repetition rules.

The main finding was that the corrected MMN (deviant minus control) was similar in the cascade and no‐repetition conditions. If the deviant in the oddball condition violates a regularity established by the standard, then the cascade condition may be preferable to the no‐repetition condition as a control condition: It has a regular sequence and does not violate any regularity, whereas the no‐repetition condition may have only an abstract rule of no repetition (Ruhnau et al., 2012). However, the data by Ruhnau et al. did not provide convincing evidence for or against this notion: A secondary Bayesian analysis of the Ruhnau et al. data provided anecdotal evidence for similar effects of cascade and no‐repetition conditions (Wiens et al., 2018). Also, in the study by Ruhnau et al., the conditions differed not only in the regularity of the tone sequence but also in number of frequencies, frequency range, and proportions of tones. These potentially confounding variables were eliminated in the present study. Furthermore, Ruhnau et al. reported that they did not find any differences between the oddball MMN (deviant minus standard) and the no‐repetition‐corrected MMN (deviant minus no‐repetition). However, the oddball MMN ought to be larger than the no‐repetition‐corrected MMN because the N1 to the standard in the oddball condition is reduced by refractoriness. Because in the Ruhnau et al. study the oddball MMN and the no‐repetition‐corrected MMN were similar, it may not be surprising that the cascade‐corrected MMN did not differ from the no‐repetition‐corrected MMN either. Simply put, if there is no difference between the oddball MMN and the no‐repetition‐corrected MMN, there is no room for the cascade condition to improve the measurement of the corrected MMN. Taken together, the present design eliminated potentially confounding effects of irrelevant variables, and the results show a clear decrease from the oddball MMN to the no‐repetition‐corrected MMN. Compared to the results by Ruhnau et al., the present results provide stronger evidence that cascade and no‐repetition rules have similar effects on the corrected MMN.

The present findings imply that both the no‐repetition rule and the cascade rule are detected as regular patterns and processed similarly. In fact, previous research has shown that the no‐repetition rule is detected as a pattern as long as repetitions occur infrequently (Horváth & Winkler, 2004; Horváth, Czigler, Sussman, & Winkler, 2001; Näätänen & Rinne, 2002; Wolff & Schröger, 2001). For example, when subjects watched a silent, subtitled movie while tones at five frequencies were presented, a statistically significant MMN was shown when the no‐repetition rule was violated on 5% of the trials but not when it was violated on 20% or 50% of the trials (Horváth & Winkler, 2004). Because violations of the no‐repetition rule were detected even though the stimulus onset asynchrony (SOA) was longer in that study than in ours (710 ms vs. 500 ms), these findings suggest that the no‐repetition rule was detected in our study.

In general, if the tone order within the control condition violates the no‐repetition rule only infrequently (<15%), this pattern is extracted as a no‐repetition rule and tone repetitions elicit an MMN within the control condition (Horváth & Winkler, 2004; Horváth et al., 2001; Näätänen & Rinne, 2002; Wolff & Schröger, 2001). This MMN may confound the measurement of the corrected MMN (deviant minus control). Accordingly, complete randomization of trials in the control condition may be problematic if the proportion of repetitions is low as a result of randomization. To address this issue, we simulated a study in which eight tones are presented 180 times (as in Ruhnau et al., 2012) but trial order is completely randomized. We found that, across all simulations (*k* = 10,000), tone repetitions would occur for a mean of 12.4% of the consecutive tones (Wiens et al., 2018). Because this percentage is low, it is likely that the no‐repetition rule is extracted and repetitions elicit a confounding MMN. To avoid this confound in the control condition, the no‐repetition rule should be applied instead of complete randomization.

We found no evidence for or against an N1 difference between cascade and no‐repetition conditions. In contrast, secondary Bayesian analyses of the Ruhnau et al. data provided very strong evidence (BF_10 _> 40) for a smaller N1 in the cascade than no‐repetition condition (Wiens et al., 2018). One potential explanation for this is that in the Ruhnau et al. study the cascade condition differed from the no‐repetition condition in other variables aside from regularity (e.g., frequency range), and these differences have been shown to decrease the N1 (Herrmann et al., 2013; Jacobsen, Schröger, et al., 2003). Controlling these aspects in the present study may have reduced the N1 differences between the cascade condition and the no‐repetition condition.

The present results have several limitations. It may be that different effects of the cascade and no‐repetition conditions on the corrected MMN would be obtained at shorter SOAs because the cascade rule ought to be extracted more easily. For example, several studies used cross‐modal oddball tasks in which the deviant occurred regularly after a fixed number of standards (e.g., four standards before the deviant) but subjects were not informed of this pattern. When the SOA was 200 ms or less, the pattern was detected, as evidenced by the absence of an oddball MMN (deviant minus standard) in the ERP (Sussman & Gumenyuk, 2005; Sussman, Ritter, & Vaughan, 1998). In contrast, when the SOA exceeded 400 ms, the pattern was undetected, as evidenced by an oddball MMN (Horacek, Kargel, Scherbaum, & Muller, 2016; Scherg, Vajsar, & Picton, 1989; Sussman & Gumenyuk, 2005; Sussman et al., 1998; Sussman, Winkler, Huotilainen, Ritter, & Näätänen, 2002). However, in MEG studies in which the pattern (i.e., three standards before the deviant) was violated over blocks (i.e., four standards before the deviant), an MMN was found to the extra standard despite an SOA of 500 ms or more (Herholz, Boh, & Pantev, 2011; Herholz, Lappe, & Pantev, 2009). Also, when the pattern was that the number of standards before the deviant increased gradually over blocks (vs. random variation over blocks), the oddball MMN (deviant minus standard) in the ERP was reduced even though the SOA was 610 ms (Lecaignard, Bertrand, Gimenez, Mattout, & Caclin, 2015). Last, an MEG study found an MMN for deviations of an eight‐tone pattern at an SOA of 500 ms (Boh et al., 2011). Taken together, these findings suggest that an SOA below 500 ms would not necessarily lead to different results. However, because previous results were mainly obtained with MEG, we cannot rule out that condition differences may be obtained with measures other than scalp‐recorded ERPs. For example, electrocorticographic recordings in an oddball task showed differences in the high‐frequency bands but not in the low‐frequency bands that are typically associated with the scalp‐recorded MMN (Durschmid et al., 2016).

Furthermore, in task conditions in which N1 and MMN tend to overlap, any condition differences in the N1 may appear as condition differences in the MMN. For example, in a study with a short SOA (200 ms), N1 and MMN tended to overlap more as the magnitude of the deviant was increased (Horváth et al., 2008). Critically, Ruhnau et al. (2012) found very strong evidence for a smaller N1 in the cascade condition than in the no‐repetition condition. Thus, in different task conditions in which N1 and MMN would overlap, the smaller N1 in the cascade condition than in the no‐repetition condition would produce a larger corrected MMN in the cascade condition than in the no‐repetition condition. However, in the study by Ruhnau et al., the cascade and no‐repetition conditions differed not only in the regularity of the tone sequence but also in number of frequencies, frequency range, and proportions of tones. As described above, these are potentially confounding variables (Herrmann et al., 2013; Jacobsen, Schröger, et al., 2003) that may have caused the condition differences in the N1. When these confounds were eliminated in the present study, no condition differences in the N1 were apparent at face value. However, the present results did not provide convincing statistical evidence for or against the idea of condition differences in N1. Therefore, more data with the present task design would be necessary to determine if the N1 differs between conditions. If it turns out that N1 differences exist, these N1 differences may appear as MMN differences in task conditions in which N1 and MMN overlap. However, our results provide statistical evidence that in a situation in which N1 and MMN do not overlap, the MMN does not differ between conditions. Therefore, a parsimonious conclusion would be that in a situation in which N1 and MMN do overlap, any condition differences reflect N1 differences rather than MMN differences.

Also, our findings may be limited by the features of the current design: It is possible that the absence of differences between the cascade and no‐repetition conditions is specific to an audiovisual cross‐modal task with simultaneous presentation of the auditory and visual input and may not extend to intramodal tasks. However, the results are relevant for typical oddball tasks in which auditory stimuli are presented while subjects perform a visual task (e.g., watching a silent movie). Further, it is unclear if our findings apply to other deviating features of auditory stimuli such as intensity, duration, and location. Although our study cannot resolve this question, parsimony suggests that if cascade and no‐repetition rules are treated similarly for frequency deviants, they would also be treated similarly for other deviating features.

Finally, the present findings do not address whether the corrected MMN reflects differences in N1 or a separate process. In the no‐repetition condition, the control tone is physically identical to the deviant in the oddball condition and is presented for the same proportion of trials as the deviant. This control condition is an important improvement over the oddball MMN (deviant minus standard) because it reduces obvious confounds from physical differences that are inherent in the oddball MMN. As such, it has been used effectively to measure a corrected MMN (deviant minus control) for frequency (Jacobsen & Schröger, 2001; Jacobsen, Schröger, et al., 2003), location (Schröger & Wolff, 1996), duration (Jacobsen & Schröger, 2003), and intensity (Jacobsen, Horenkamp, et al., 2003). Initially, the corrected MMN was referred to as the genuine MMN and was believed to eliminate confounding N1 effects. However, this question continues to be a matter of debate (Fishman, 2014). Indeed, some authors argue that the corrected MMN is merely a difference in N1 that is caused entirely by neural adaptation (May & Tiitinen, 2010). If so, the term MMN is a misnomer if it is used to imply a neural mechanism that is separate from the N1. Nonetheless, irrespective of its actual neural mechanism, the apparent frontocentral negativity between the deviant from the oddball and the control tone from either cascade or no‐repetition condition apparently captures the violation of a regularity (Winkler, Denham, & Nelken, 2009).

To conclude, the cascade condition may have an intuitive appeal: With its up‐and‐down sequence, it seems to have a clear regularity compared to the no‐repetition condition. However, the preregistered analyses provided moderate evidence (BF_01 _> 6) that cascade and no‐repetition conditions have similar effects on the corrected MMN (deviant minus control). These findings are consistent with previous research that the no‐repetition rule is detected by the auditory system. Further, they imply that the no‐repetition rule is processed similarly to the cascade rule. Accordingly, the no‐repetition condition provides an adequate control for the oddball condition in frequency MMN.
